# Differences in playing style and technical performance according to the team ranking in the Spanish football *LaLiga*. A thirteen seasons study

**DOI:** 10.1371/journal.pone.0293095

**Published:** 2023-10-20

**Authors:** Joaquín González-Rodenas, Jordi Ferrandis, Víctor Moreno-Pérez, Roberto López-Del Campo, Ricardo Resta, Juan Del Coso

**Affiliations:** 1 Sport Sciences Research Centre, Rey Juan Carlos University, Fuenlabrada, Spain; 2 Catholic University of Valencia, “San Vicente Mártir”, Valencia, Spain; 3 Sports Research Center, Miguel Hernandez University of Elche, Alicante, Spain; 4 Department of competitions and Mediacoach, LaLiga, Madrid, Spain; Instituto Politécnico de Santarém: Instituto Politecnico de Santarem, PORTUGAL

## Abstract

This study aimed to explore the differences in playing style and technical performance according to the ranking level in Spanish football teams. The sample comprised 38 professional teams that competed in *LaLiga* from 2008/09 to 2020/21, with a total of 4940 matches. The teams were grouped by their final ranking position: Champion (1st); Champions League (2^nd^–4^th^); Europa League (5^th^–6^th^); middle teams (7^th^–17^th^); and relegated teams (18^th^–20^th^). Linear mixed models were used to examine the effects of the team ranking on variables related to playing style and technical performance. The F^2^ statistic was calculated as effect size (ES). Regarding the style of play, the Champion teams initiated offensive sequences from a more advanced field position than the remaining ranking groups with a descending effect as the ranking position decreased (p < 0.001; ES = medium). The sequence duration and passes per sequence showed a decreasing effect across ranking groups (both p < 0.001; ES = small). In contrast, the direct speed showed an increasing effect as the ranking position decreased (p < 0.001; ES = small). A decreasing effect was observed in the number of sequences ending in the final third as the ranking position decreased (p < 0.001; ES = large) while no effect was found for the sequences ending in a shot. There was a reduction effect in ball possession, passing accuracy, through balls and crosses as the teams’ ranking decreased (all p < 0.001; ES = small-to-large). In summary, higher-ranked teams had an advanced starting position, longer offensive sequences, slower progression speed, more ball possession, higher passing accuracy, and more crosses and through balls than lower-ranked teams. Football coaches should consider that adopting a playing style focused on regaining the ball possession in advanced field zones and using long passing sequences can be an effective tactical strategy to improve the teams’ ranking during the season.

## Introduction

Football (soccer) is a field game played by two opposing teams where players compete directly, indirectly, and simultaneously to achieve an objective that involves scoring goals. At the same time, each team works to prevent the opposing team from scoring, which requires complex interaction and coordination among all players to achieve these specific ends [1]. In this regard, the analysis of teams’ technical performance and playing style in football is exponentially growing in the last decade [2]. Currently, the amount of match data obtained in professional football is not only larger than several years ago but also more accurate so that it captures more representative performance indicators [3]. In this regard, there has been an evolution from the collection of discrete performance indicators such as number of passes, shots, or dribbles per player towards more complex metrics related to the space-time interactions between teams and emerging playing configurations [4]. With this information, team coaches can assess the individual or collective performance during competition, which can help them to design more precise game plans and training sessions [5, 6].

Despite the complex and multifactorial nature of football, some recent studies have explored the main technical differences between successful and unsuccessful teams during matches, tournaments, and domestic competitions [7]. In general, existing literature has agreed that high-ranked teams have higher shooting quantity and accuracy, as well as higher values of ball possession and passing accuracy than low-ranked teams in national leagues from Spain [8], Germany [9, 10], Greece [11] and China [12, 13], as well as in different World Cups [14–16]. For instance, Casal et al. [17] observed that the best teams in the Spanish *LaLiga* performed higher number of successful passes, while the bottom teams executed more defensive than offensive actions. Similarly, Fernández-Cortes et al. [18] revealed that Spanish teams ranked in a European competition position reported a higher ball possession and total number of passes. In the same competition, Brito da Souza et al. [19] highlighted that the match statistics that best discriminated successful from unsuccessful football teams were shooting accuracy and the number of shots conceded to the opposing team. Collectively, all this information suggests that the technical performance of higher-ranked teams is superior to lower-ranked teams, which is probably a cause of their success.

In addition to the technical performance, recent studies have also evaluated collective playing styles by quantifying elements such as the speed of the ball when progressing to the opponent’s goal, the duration and number of skilled actions, and the location of ball possession and player’s movements [20]. In this sense, the existing literature suggests that high-ranked teams implement a more combinative playing style with higher ball possession [11, 21], more game initiative [22], and a reduced use of direct playing styles [23], in comparison with low-ranked teams. In the Spanish *LaLiga*, recent studies observed that implementing a style of play based on elaborate attacks with a high quantity of passes per possession was associated with winning [24]. Also, Casal et al [17] observed that the higher-ranked Spanish teams performed a greater number of dynamic offensive transitions than the bottom-ranked teams. Therefore, it seems that a combinative playing style with more passes per sequence may favor football performance, but more information is needed to determine whether this potentially favorable playing style is beneficial irrespective of the match location or the quality of the opposing team.

To this regard, the multifactorial nature of football performance requires considering that the technical performance and playing style of teams can be strongly influenced by contextual variables such as match location, quality of the opposition and match outcome [25, 26]. For example, home teams display a more offensive style of play [27] with a more advanced defensive position on the field [28] than when playing away. In this sense, this higher offensive predisposition may be due to greater winning expectative and a more offensive strategy when playing at home [29]. Regarding the quality of the opposition, Fernandez-Navarro et al. [25] observed an increase in direct play and decrease in variables such as build up, maintenance, crossing, fast tempo and high pressure when playing against stronger opposition. In the same line, previous studies have observed that playing against weak opposition was associated with displaying higher offensive length, width and surface area [30, 31] and more ball possession [32, 33].The match outcome also plays an important role in the technical performance and playing style of football teams. In fact, recent studies have observed that winning teams show higher offensive production, shot conversion and ball possession than losing teams [15, 34].

In this sense, despite the interesting results provided by previous research studies, the use of large datasets that analyze the teams´ performance across different competitive seasons can provide a more depth understanding of the interaction between technical and tactical indicators and teams’ quality. In addition, most previous studies used comparative or correlation analyses without considering the combined effects of contextual variables, what is key to understand the complexity of teams’ performance. Besides this, existing studies have compared high-ranked versus low-ranked teams according to two, three or four different position groups, while the European leagues already have a specific organization of the rankings considering different levels of success according to the achieved position (*i*.*e*., qualification for UEFA Champions League, for Europa League or relegation to the second league), which distinguish the prestige and status of clubs at the end of the season. Thus, exploring the technical and tactical differences between specific standing positions could help identify the key indicators that distinguish the teams ‘performance according to the achievement of different levels of success.

Therefore, the aim of this investigation was to explore the differences in playing style and technical performance variables according to the ranking level in Spanish *LaLiga* football teams, considering the effects of match location, quality of opposition and match outcome. Based on previous studies performed in the Spanish *LaLiga* [8, 17–19, 24] we hypothesized that higher-ranked teams would implement more combinative and productive team sequences, which involve longer offensive sequences with more passes, more dribbling and a greater number of through balls, crosses, key passes, shots and shooting accuracy, than lower-ranked teams.

## Methods

### Sample

The sample included 38 professional football teams that competed in the first division of Spanish football (*LaLiga*) from the season 2008/09 to 2020/21, playing a total of 4940 matches. All matches played during the thirteen seasons under investigation were included in the study regardless of player dismissals or other possible contextual factors. The teams were grouped according to the ranking position at the end of the season and using the following clusters: the league Champion (1^st^; n = 494 matches; 5%); teams classified for the Champions League (2^nd^–4^th^; n = 1482 matches; 15%); teams classified for the Europa League (5^th^ and 6^th^; n = 988 matches; 10%); teams in the middle of the ranking (7^th^–17^th^; n = 5434 matches; 55%); and the relegated teams (18^th^–20^th^; n = 1482 matches; 15%), following previous investigations [22, 35].

Data were provided by *LaLiga*, which authorized the analysis of the variables included in this investigation and the publication of results with a scientific objective. Written informed consent was obtained from all the participants and teams involved in this investigation. Due to the ethical guidelines of *LaLiga*, this investigation does not include information that identifies football players, and it has been treated in accordance with the Declaration of Helsinki. The Institutional Review Board of the Rey Juan Carlos University approved this study.

### Match analysis

This investigation is a descriptive and comparative analysis to study the characteristics of the offensive sequences in professional football teams according to their competition level, assessed by the ranking position obtained at the end of each season. Operationally, an offensive sequence was defined as a passage of play that belongs to one team that ended by a defensive action, any kind of stoppage in play, or a shot. The characteristics of each offensive sequence were obtained for each of the 38 matches that were played in each season.

For each team under investigation, eight variables related to the playing style during the offensive sequences were evaluated. These variables included: 1) sequence start (the location at the pitch where the sequence started, in meters, with respect to the team’s own goal); 2) sequence duration (time of the sequence, in s), 3) passes per sequence (number of passes per sequence); 4) direct speed (the distance the ball moved towards opposing team’s goal line during the sequence per second, in m/s), 5) sequence width (distance between the leftmost point and the rightmost point reached by the ball in the sequence, in m); 6) sequence length (the total distance the ball travelled during the sequence, in m) 7) sequences that end in the final third (number of sequences that ended in the attacking third of the field) and 8) sequences that end in a shot (number of sequences that created a shot).

Additionally, other eight offensive technical variables were included in this study such as: 1) ball possession (percentage of match play time that the team had the ball); 2) passing accuracy (percentage of passes completed successfully); 3) crosses (ball played from a wide position intending to reach a teammate in a specific area in front of the opponent’s goal); 4) through balls (a pass that overcomes the rival’s defence towards the opposing goal); 5) dribbles (attempt by a player to beat an opponent when he has possession of the ball), 6) dribbling accuracy (percentage of successful dribbles); 7) key passes (the final pass leading to the recipient of the ball having a goal attempt) and 8) shot conversion (calculation of goals scored divided by shots attempted, in percentage).

These specific variables were selected for this study because they capture representative information to analyze the playing style implemented by teams, such as the start, development, progression, and the offensive production of the tactical sequences [11, 20].

### Procedure

All these variables were recorded in each football match individually for each team competing by a valid and reliable video tracking system that assesses teams’ match statistics during match play (*i*.*e*., Mediacoach®) [36]. This video tracking system uses information from OPTA Sportsdata and then organizes and exported the information to facilitate the analysis of variables for professional football teams [37]. Previous works have presented the analysis of several of these data such as match statistics [8, 38, 39] and offensive and defensive playing style variables [22]. With this system of data offered, all the sequences produced in each *LaLiga* match were automatically recorded and stored. Afterwards, each offensive sequence can be classified according to the characteristics described above.

### Statistical analysis

The data were transferred from Mediacoach to a .csv database which was organized and exported into Microsoft Excel. All the variables were collected for the two opposite teams competing in each match, and an average was obtained for each variable in each match (*i*.*e*., there were 38 data per team and per season for each variable explained above). Then the groups were created by allocating each team to the corresponding cluster, depending on the ranking position they had obtained at the end of the season, considering previous studies: (1) Champion; (2) Champions League; (3) Europa League; (4) Middle; (5) Relegation. The amount of data per season in each group varied depending on the number of teams in each group. For example, in any of the thirteen seasons under investigation, there were 38 datasets for the Champion (38 matchdays × 1 team in the group) and 418 datasets per season for the group categorized as middle ranking (38 matchdays × 11 teams in the group). The Microsoft Excel file was then exported to SPSS (IBM, Version 27.0, USA). The assumption of normality of the data was confirmed graphically and using the Kolmogorov–Smirnov test and the homogeneity of variances was examined and confirmed using the Levene’s test.

Due to the hierarchical structure of teams’ performance in football (each team has its own playing style), a multilevel mixed model [40] was performed to cluster the collective performance (level 2) into teams (level 1). With this organization of the data, a generalized linear model was carried out to explore the effects of the team ranking (fixed effects) on the multiple tactical and technical variables, considering the effect of the team (random effects). The regression model was used to estimate the means (i.e., expected value) and its confidence interval for each group of data by plugging the real value into the fitted model equation. To produce a more solid exploration, we have considered the effect of several contextual variables that have been shown to have a relevant significancy in match technical and tactical performance [23, 25] such as match location (home vs away), opponent team’s quality in quartiles (first quartile, second quartile, third quartile and fourth quartile) and match outcome (win, draw and loss). The Cohen F^2^ statistic was calculated as the effect size (ES) of the fixed effects [41]. The Cohen F^2^ effect size is a measure of the proportion of variance in the outcome explained by the fixed effects included in the model. In this regard, it was considered a trivial effect at a value lower than 0.02, small at a value of 0.02, medium at a value of 0.15 and large at a value of 0.35 [42].

Finally, graphic charts with the estimated means and confidence intervals were displayed. For these variables, pairwise comparisons of the estimated means were performed through Fisher’s Least Significant Difference Tests (LSD). The significance level was set to p < 0.050.

## Results

**Fig 1:**
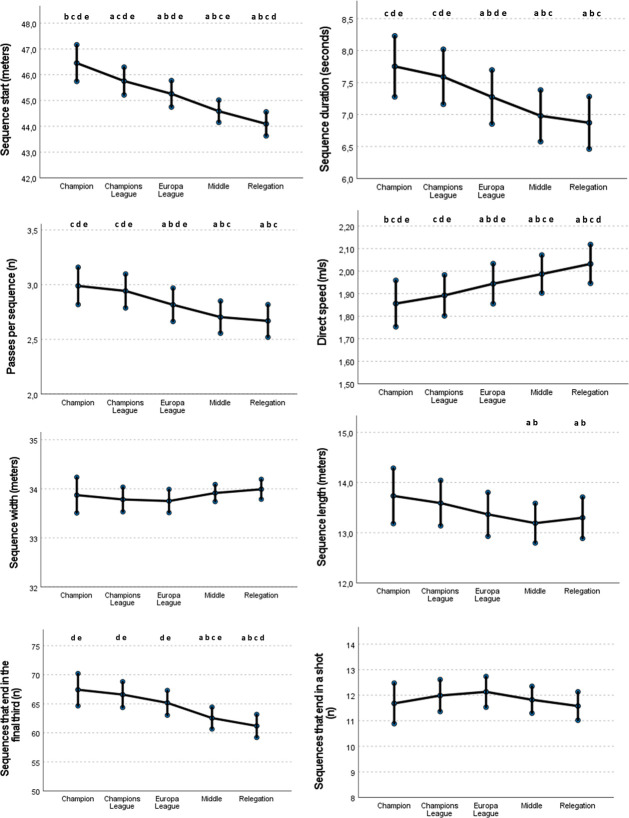
Estimated means and confidence intervals of playing style variables according to the team’s ranking at the end of the season in *LaLiga* from 2008/09 to 2020/21 after adjusting for contextual variables. **a** *= different from the Champion teams;* **b** *= different from the Champions League teams;* **c** *= different from Europa League teams;* **d** *= different from Middle teams;* **e** *= different from Relegation teams*.

Table 1 depicts the comparative analysis of playing style and technical performance variables according to the teams’ ranking considering the effects of match location, quality of the opposition and match outcome. Regarding the variables related to the playing style, the Champion teams started the offensive sequences in a more advanced position than the remaining ranking groups, showing a statistically significant and moderate reduction of the position where the sequence was started as the ranking position decreased (Champions League teams: Coeff = -0.604, SE: 0.25, p < 0.01; Europa League teams: Coeff = -1.06, SE: 0.32, p < 0.01; middle teams: -1.773, SE: 0.31, p < 0.001; relegation teams: Coeff = -2.271, SE: 0.33, p < 0.001), with statistically significant differences among all the ranking groups (Fig 1).

**Table 1:** Multilevel mixed multivariate model to predict the playing style and technical performance variables according to teams’ ranking at the end of the season in *LaLiga* from 2008/09 to 2020/21. The multivariate model includes the effect of match location, quality of the opponent and match outcome. *≤ 0.05; ** ≤ 0.01; *** = 0.001.

	Variable (units)	Intercept	Effect Size	Champion teams	Champions League teams	Europa League teams	Middle teams	Relegation teams
		Coeff (SE)			Coeff (SE)	Coeff (SE)	Coeff (SE)	Coeff (SE)
**Playing style**	Sequence start (m)	41.16 (0.42)***	Medium	Reference	-0.604 (0.25)**	-1.06 (0.32)**	-1.773 (0.31)***	-2.271 (0.33)***
	Sequence duration (seconds)	6.92 (0.26)***	Small		-0.144 (0.11)	-0.458 (0.11)**	-0.751 (0.13)***	-0.856 (0.15)***
	Passes per sequence (n)	2.71 (0.09)***	Small		-0.40 (0.03)	-0.166 (0.04)**	-0.278 (0.05)***	-0.312 (0.05)***
	Direct speed (m/s)	1.970 (0.06)***	Small		0.033 (0.02)	0.084 (0.03)*	0.128 (0.03)***	0.174 (0.03)***
	Sequence width (m)	33.68 (0.22)***	Small		-0.085 (0.14)	-0.109 (0.18)	0.047 (0.177)	0.116 (0.19)
	Sequence length (m)	12.68 (0.31)***	Small		-0.124 (0.17)	-0.348 (0.22)	-0.520 (0.21)*	-0.399 (0.23)*
	Sequences that end in the final third (n)	44.56 (1.61)***	Large		-0.363 (0.91)	-1.677 (1.16)	-4.437 (1.11)***	-5.794 (1.21)***
	Sequences that end in a shot (n)	7.164 (0.46)***	Trivial		0.390 (0.27)	0.544 (0.33)	0.213 (0.32)	-0.024 (0.35)
**Technical performance**	Ball possession (%)	44.06 (1.01)***	Small		-0.709 (0.51)	-1.992 (0.64)**	-3.647 (0.62)***	-5.596 (0.68)***
	Passing accuracy (%)	74.82 (0.83)***	Small		-0.953 (0.38)	-2.098 (0.49)***	-3.108 (0.48)***	-3.316 (0.52)***
	Crosses (n)	13.03 (0.82)***	Large		-0.820 (0.47)	-2.164 (0.59)***	-2.249 (0.58)***	-3.351 (0.62)***
	Through balls (n)	1.97 (0.28)***	Small		-0.323 (0.15)*	-0.395 (0.19)*	-0.428 (0.18)*-	-0.798 (0.20)***
	Dribbles (n)	15.20 (0.74)***	Small		0.525 (0.38)	0.300 (0.49)	0.371 (0.47)	1.452 (0.51)**
	Dribbling accuracy (%)	45.06 (1.55)***	Small		0.795 (0.92)	-1.045 (1.17)	-2.02 (1.1)*	-2.89 (1.22)*
	Key passes (n)	4.47 (0.36)***	Small		0.459 (0.21)	0.388 (0.27)	0.213 (0.26)	0.073 (0.28)
	Shot conversion (%)	12.51 (0.70)***	Medium		-0.494 (0.51)	0.384 (0.59)	-0.042 (0.53)	-0.052 (0.58)

Other variables such as the sequence duration and passes per sequence also showed a small decreasing effect across the ranking groups, so that the Champion teams performed more passes per sequence and implemented longer passing sequences that the remaining ranking positions (Table 1). In fact, statistical differences were found among all groups, except between the Champion teams and the teams that qualified for the Champions League (Fig 1) for these two variables. In contrast, the direct speed of progression showed a small increasing effect as the ranking position decreased showing statistical differences among all groups (Fig 1) except for the Champions League teams’ group which was not different from the Champion teams. In this sense, Europa league teams (Coeff = 0.084, SE:0.03; p < 0.01), Middle teams (Coeff = 0.128, SE:0.03; p < 0.001) and the Relegation teams (Coeff = 0.174; SE = 0.03; p < 0.001) progressed faster than the Champion teams.

As for the length and width of the offensive sequences, the Champion teams registered significantly more length than the Middle teams (Coeff = -0.520; SE = 0.31; p < 0.05) and Relegation teams (Coeff = -0.399; SE = 0.23; p < 0.05). On other hand, the Champion teams, Champions League teams and Europa League teams showed less offensive width than Middle and Relegation teams, although these differences were not statistically significant.

Regarding the penetration of the offensive sequences, the Champion teams ended more sequences in the final third than the Middle-ranking teams (Coeff = -4.437; SE:1.11; p < 0.001) and the Relegation teams (Coeff = -5.794; SE: 1.21; p < 0.001), although no differences were found among ranking groups for the number of sequences that ended in a shot.

**Fig 2:**
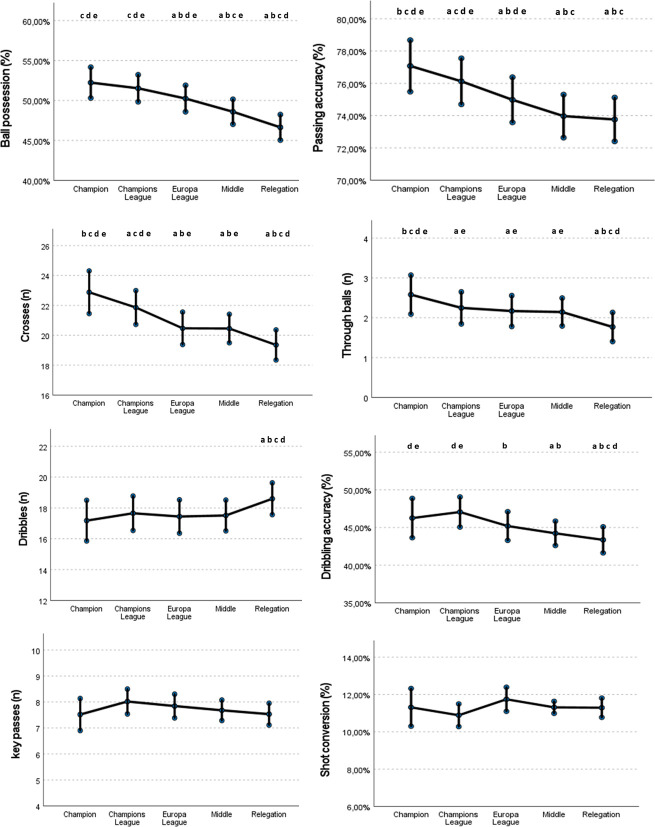
Estimated means and confidence intervals of offensive technical performance variables according to the team’s ranking at the end of the season in *LaLiga* from 2008/09 to 2020/21 after adjusting for contextual variables. **a** *= different from the Champion teams;* **b** *= different from the Champions League teams;* **c** *= different from Europa League teams;* **d** *= different from Middle teams;* **e** *= different from Relegation teams*.

As for the technical variables, there was a small reduction of the percentage of ball possession, passing accuracy and through balls, as well as a large reduction in the number of crosses as the teams’ ranking position decreased (Fig 2). This descending effect was statistically significant for all the groups in comparison with the Champion Teams.

No statistical differences were found among ranking groups in relation to the number of dribbles per game, except for the Relegation teams that showed more dribbles than the Champion teams (Coeff = 1.452; ES = 0.51; p < 0.05). However, the Relegation teams (Coeff = -2.89; ES = 1.22; p < 0.001) and the Middle-ranking teams (-2.02; ES = 1.10; p < 0.01) presented a statistically significant lower percentage of dribbling accuracy than the Champion, with a small size effect.

Finally, no statistical differences were observed among ranking groups in terms of key passes and shot conversion ratio.

## Discussion

This study aimed to explore the differences in playing style and technical performance variables according to the team’s ranking in a large sample of matches of the first division in Spanish football (*LaLiga)*. Our study revealed meaningful although generally small differences in team’s playing style and technical performance variables among the multiple ranking levels established for this investigation, especially between the Champion teams and all the remaining ranking groups.

Regarding the start of the offensive sequences, the Champion teams initiated the sequences from more advanced zones on the field than the rest of the ranking groups, with a descending trend as the ranking group decreased. Previous studies found that stronger teams employed more proactive defensive strategies [43, 44], as well as they were more effective when applying defensive pressure in more advanced zones [45]. For instance, the study by Volgelbein et al. [46] observed that top teams in the German *Bundesliga* regained ball possession significantly faster than the less successful teams and demonstrated lower defensive reaction times. In the same competition, successful teams tend to use the counterpressing strategy more efficiently [47], what indicates that recovering the ball in advanced zones of the field seems very important to achieve better offensive performance. In this sense, data of the current investigation revealed that the most successful teams in *LaLiga* seemed to be able to regain the ball possession further from their own goal, which creates an important tactical advantage, so that the team initiates the offensive sequence closer to the opposing goal.

As for the development of the offensive sequences, higher-ranked teams registered more passes per sequence and longer duration than lower-ranked teams. Our study also found a descending trend in ball possession, passing accuracy and dribbling accuracy as the ranking position decreased. These findings revealed that top-placed teams seem to adopt a more possession-oriented style of play, as previous studies observed in several European domestic competitions [9, 11, 48–50]. As it is expressed in our study, this possession-oriented style of play seems effective to obtain the title in *LaLiga*, as the Champion was the team with the highest values in all these variables, despite there were three different teams that were included in this group for the thirteen seasons under investigation (Atlético de Madrid was Champion in two seasons, Barcelona in eight seasons and Real Madrid in three seasons). Surprisingly, no differences were found regarding the sequence’s width between the Champion and all the remaining ranking groups. Previous studies had highlighted the importance of increasing the dispersion in width of players during the offensive phase, so that adopting a wider pitch space occupation favors ball possession [51] and contributes the creation and occupation of more open spaces [52]. However, our study suggests that the width of the teams’ offensive sequences was not a tactical aspect that differentiated the teams’ ranking at the end of the season.

A key finding of our study was that the speed of progression was significantly lower for the higher-ranked teams. Specifically, the direct speed showed a slightly progressive increase from the Champion to the Relegated teams (Fig 1). This fact highlights that the higher-ranked teams had more patience to progress, likely moving the ball more horizontally, using longer sequences and waiting for the right moment to break opposing lines, regardless of match location, quality of the opponent and match outcome. Contrarily, lower ranked teams may use more frequently counterattacks or fast attacks by using quick changes of the point of attack to advance faster to the opposing goal. These playing styles in lower-ranked teams would explain the higher speed of progression and reduced ball possession, but also the lack of differences in the sequence’s width in comparison with the higher-ranked teams.

However, despite the top-ranked teams showed slowest speed to gain space vertically, these teams progressed more meters per sequence (as expressed as the sequence length) than teams in lower ranking positions. This more advanced offensive progression of better-ranked teams was not only observed considering the number of meters advanced but also registering more entries into the final third. All these parameters decreased as the position in the ranking was lower, although the main differences were found between the teams that qualified for European competitions (Champion, Champions League teams and Europa League teams) in comparison with the middle-ranked and the teams that relegated to the Spanish second division. Collectively, all this information suggests that most successful teams employ longer and more horizontal sequences that are more effective that the use of a vertical style of play of lower-ranked teams as these longer sequences obtain a better progression and a higher probability of reaching the attacking third.

Furthermore, the most successful teams registered more crosses and through balls than lower-ranked teams, although no differences were found in relation to the number of key passes among the ranking position groups. These findings reflect a higher penetrative activity into the opposing box of the better-ranked teams than the rest of the ranking groups. In this regard, crosses and through balls may emerge when high ranked teams attempt to break high compact defenses positioned in a low block structure, what promotes the use of these attacking strategies to penetrate the box. On one hand, some studies in different competitions [15, 53] found that the probability of winning increased when teams searched for scoring opportunities with penetrative passes (through balls) rather than crossing the ball from wide areas. This negative impact of crossing on the offensive team performance has also been reported in different studies [54, 55], which suggested that the rate of ineffective crosses greatly exceeds the rate of effective crosses. For example, the study by Lago-Peñas et al. [34] observed that winning teams performed a lower number of crosses but conceded a higher number of crosses. In the same line, Zhou et al., (2020) [56] found that the teams performed more crosses when losing than when winning in the Chinese Super League. However, a recent study by Casal et al. [17] observed that a greater number of crosses [57] and effective crosses were among the most important factors to achieve offensive success in *LaLiga* football teams, what is aligned with our findings. Likewise, a very recent study in the German *Bundesliga* [9] found a positive correlation between the number of crosses and the points achieved at the end of the season. Thus, despite the controversial results of different studies, the authors of this study consider that crossing can be a useful strategy in football as its frequency was higher in high-ranked teams in the present study. However, more research seems to be necessary to clarify the tactical effectiveness of crossing in different contexts, as its utility may vary depending on the characteristics of the teams or the attributes of each football competition [58, 59].

In relation to the finishing process of the offensive sequences, no differences were found in relation to the number of shots and shot conversion ratio. These results are surprising because they are not in line with multiple previous studies that had observed that the main differences between successful and unsuccessful teams were based on the number of shots and shot accuracy [8–11, 13, 14, 60]. For instance, Casal et al [17] revealed that effective shots were one of the key factors related to success in the Spanish *LaLiga* football teams. Similarly, the study by González-Rodenas et al. [23] observed that the higher-ranked teams from the top four European leagues had higher shooting effectiveness than the low-ranked teams of these competitions, regardless of the effect of different defensive, offensive or contextual variables. Another recent study by Andrzejewski et al. [9] revealed that the number of shots on goal registered a very strong correlation with the total points earned by teams at the end of the season in the German Bundesliga. These different findings are probably due to the methods used to analyze the teams’ performance among studies. In this regard, the present study considered within the model the influence of match location, quality of opposition and match outcome to analyze the effects of team ranking on playing style and technical performance while most previous studies used comparative or correlation analysis without taking into account the effects of key contextual variables that have been demonstrated to have a strong effect of team performance [23, 25, 26]. In this regard, while the univariate effect of team ranking on shot conversion could show that higher ranking teams have higher percentage accuracy with a large size effect (supplementary material, Table 2), this effect was minimized when adjusting by contextual variables.

**Table 2:** Multilevel mixed univariate model to predict the playing style and technical performance variables according to teams’ ranking at the end of the season in *LaLiga* from 2008/09 to 2020/21. *≤ 0.05; ** ≤ 0.01; *** = 0.001.

	Variable (units)	Intercept	Effect Size	Champion teams	Champions League teams	Europa League teams	Middle teams	Relegation teams
		Coeff (SE)			Coeff (SE)	Coeff (SE)	Coeff (SE)	Coeff (SE)
**Playing style**	Sequence start (m)	46.051 (0.37)***	Large	Reference	-0.513 (0.28)	-0.893 (0.35)*	-1.373 (0.34)***	-1.609 (0.36)***
	Sequence duration (seconds)	7.683 (0.24)***	Small		-0.129 (0.11)	-0.409 (0.14)**	-0.672 (0.14)***	-0.746 (0.15)***
	Passes per sequence (n)	2.959 (0.08)***	Trivial		-0.032 (0.03)	-0.144 (0.05)**	-0.243 (0.05)***	-0.262 (0.05)***
	Direct speed (m/s)	1.863 (0.05)***	Small		0.032 (0.03)	0.078 (0.03)*	0.117 (0.03)***	0.155 (0.03)***
	Sequence width (m)	33.904 (0.18)***	Small		-0.102 (0.14)	-0.148 (0.18)	0.001 (0.17)	0.059 (0.19)
	Sequence length (m)	13.626 (0.28)***	Small		-0.097 (0.17)	-0.283 (0.22)	-0.413 (0.21)	0.262 (0.23)
	Sequences that end in the final third (n)	65.945 (1.47)***	Small		-0.171 (1.03)	-1.290 (1.31)	-3.250 (1.25)*	-3.670 (1.47)**
	Sequences that end in a shot (n)	11.847 (0.42)***	Medium		0.271 (0.95)	0.353 (0.36)	-0.044 (0.34)	-0.334 (0.37)
**Technical performance**	Ball possession (%)	51.540 (1.01)***	Small		-0.341 (0.56)	-1.477 (0.71)*	-2.871 (0.69)*	-4.357 (0.75)***
	Passing accuracy (%)	76.676 (0.81)***	Small		-0.759 (0.40)	-1.702 (0.51)**	-2.508 (0.49)***	-2.473 (0.53)***
	Crosses (n)	21.506 (0.76)***	Small		-0.461 (0.52)	-1.490 (0.66)*	-0.945 (0.64)	-1.330 (0.69)
	Through balls (n)	2.691 (0.25)***	Small		-0.366 (0.15)*	-0.472 (0.19)*	-0.551 (0.18)**	-0.976 (0.20)***
	Dribbles (n)	17.123 (0.67)***	Small		0.505 (0.38)	0.314 (0.49)	0.404 (0.47)	1.523 (0.51)**
	Dribbling accuracy (%)	46.548 (1.33)***	Small		0.711 (0.92)	-1.226 (1.17)	-2.331 (1.12)*	-3.331 (1.21)**
	Key passes (n)	7,406 (0.31)***	Small		0.561 (0.22)*	0.403 (0.28)	0.284 (0.27)	0.219 (0.29)
	Shot conversion (%)	16.130 (0.60)***	Large		-2.258 (0.60)***	-3.052 (0.70)^***^	-5.277 (0.61)***	-7.039 (0.66)***

Finally, it is important to highlight how this investigation consistently found tactical and technical differences between the Champion and the rest of the ranking groups assessed, except for the teams qualified for the Champions League. Particularly, no differences were found in fourteen out of sixteen variables between the Champion and the teams that qualified for the Champions League, which suggests that the higher-ranked teams share a similar playing style with comparable offensive technical performance and only subtle differences in these variables are responsible of the final ranking obtained by the teams.

This study contains limitations that require acknowledgment. First, all the variables evaluated in this research are related to the offensive process, which limits the capacity to capture the complex dynamic of football tactics, based on both offensive and defensive moments. Further, the current investigation has been performed with data from a national football league of professional male players and the results should not be extrapolated to other leagues, other categories or women’s football. However, the large sample of teams analyzed in this study reinforces the consistency of the findings, which have important practical applications.

From a practical standpoint, these findings provide new tactical and technical insights for football coaches and practitioners. In this regard, using a playing style that regains the ball possession in advanced zones of the field and that implements long passing sequences seems to be a positive tactical approach to increase the team’s success during the season. Also, football coaches and practitioners should consider the importance of performing a high quantity of through balls and crosses as a part of the playing style, which may require specific training exercises during coaching sessions.

In conclusion, higher-ranked teams in the Spanish *LaLiga* implemented offensive sequences that: 1) started in more advanced zones of the field; 2) included more passes and longer duration; 3) presented slower direct speed to progress towards the opposing goal; but 4) advanced more meters through the field, than the lower-ranked teams. Regarding technical variables, higher-ranked teams registered: 1) higher ball possession; 2) higher passing accuracy; 3) higher dribbling accuracy, 4) more through balls and 5) more crosses than lower-ranked teams.
